# Definition and attributes of the emotional memory images underlying psychophysiological dis-ease

**DOI:** 10.3389/fpsyg.2022.947952

**Published:** 2022-11-14

**Authors:** Matt Hudson, Mark I. Johnson

**Affiliations:** ^1^Mind Help Limited, Durham, United Kingdom; ^2^Centre for Pain Research, Leeds Beckett University, Leeds, United Kingdom

**Keywords:** emotional memory image, psychophysiological dis-ease, psychological trauma, Split-second Unlearning, psychological therapies focused on trauma, freeze response, preverbal trauma, hypothalamic-pituitary-adrenal axis

## Abstract

**Background:**

Previously, we proposed a “*Split-second Unlearning*” model to explain how emotional memories could be preventing clients from adapting to the stressors of daily living, thus forming a barrier to learning, health and well-being. We suggested that these emotional memories were mental images stored inside the mind as ‘emotional memory images’ (EMIs).

**Objective:**

To elaborate on the nature of these emotional memory images within the context of split-second learning and unlearning and the broader field of psychoanalysis, to initiate a conversation among scholars concerning the path that future healthcare research, practice, and policy should take.

**Method:**

A narrative review of the attributes of EMIs utilizing relevant and contentious research and/or scholarly publications on the topic, facilitated by observations and approaches used in clinical practice. Results: We propose a refined definition of EMIs as *Trauma induced, non-conscious, contiguously formed multimodal mental imagery, which triggers an amnesic, anachronistic, stress response within a split-second*. The systematic appraisal of each attribute of an EMI supports the idea that the EMI is distinct from similar entities described in literature, enabling further sophistication of our Split-second Unlearning model of psychophysiological dis-ease.

**Conclusion:**

Exploration of the concept of EMIs provides further insight on mechanisms associated with psychophysiological dis-ease and opportunities for therapeutic approaches.

## Highlights

- Emotional memory images are mental images stored inside the mind and distinct from other psychological entities described in literature.
- Re-triggering emotional memories in daily living leads to recurrent stress and persistent psychophysiological dis-ease.
- Exploration of the concept of EMIs offers therapeutic opportunities.

## Introduction

Previously, we proposed a model describing the psychophysiological stress responses that occur whenever individuals encounter situations that trigger, within a very short time frame, an emotional memory associated with previous traumatic event(s), i.e., “*split-second learning*” (Hudson and Johnson, 2021). We suggested that, once formed, these emotional memories can be frequently re-triggered in daily living, leading to the re-playing of psychophysiological stress responses and a state of persistent psychophysiological dis-ease.

We described how these emotional memories that were learned in a split-second could be preventing clients from adapting to the stressors of daily living, thus forming a barrier to health and well-being. We offered a novel therapeutic approach called ‘Split-second Unlearning’ that involved the surveillance of clients for non-conscious ‘freeze-like’ micro-expressions that may signify the presence of an ‘in-the-moment’ stress response associated with the emotional memory. The client was then encouraged to become a curious observer within/of their own experience, feeding back the non-verbal cues as they arrive in the moment. We argued that breaking down and interrupting the informational flow of observable fragments within a client’s split-second Pavlovian response enables clients to detach their emotional memory from the psychophysiological stress response. We argued that a major advantage of the Split-second Unlearning approach is that the client remains at the center of the ‘therapeutic intervention,’ without the need to get bogged down in a whole-life narrative.

Emotional memories are central to our Split-second Unlearning model. We suggested that these emotional memories were mental images stored inside the mind as ‘emotional memory images’ (EMIs) that were non-conscious and intrinsically linked to a set of physical reactions, including eye movement and/or fixation (Hudson and Johnson, 2021).

The purpose of this article is to elaborate on the nature of these emotional memory images within the context of split-second learning and unlearning and the broader field of psychoanalysis. We have structured the review to provide a brief overview of the attributes of EMIs, followed by more detailed discussion of:

• Instigating the concept.
• Working definition.
• Attributes.
• What EMIs confer on humans including;
  ⚬ Evolutionary perspectives,
  ⚬ How EMIs affect humans in positive and negative ways,
  ⚬ Relationship to psychophysiological dis-ease,
  ⚬ How we can interact with them in a therapeutic context, and
  ⚬ Research conducted to date.

Our narrative utilizes relevant and contentious research and/or scholarly publications on the topic. We acknowledge that this non-systematic approach is open to selection biases and opinion-driven arguments, and therefore we emphasize that the purpose of this article is to initiate a conversation among scholars concerning the path that future healthcare research, practice, and policy should take.

## Emotional memory image: Instigating the concept

Our thinking about the concept of an EMI and its association with psychophysiological dis-ease arose from a clinical observation made by MH whilst working with a client who presented with a wasp phobia. During conversation, MH noticed that the client’s eyes would lock momentarily on a specific area within her visual field. This non-verbal response seemed to suggest that the client was momentarily ‘accessing her phobia.’ The client was unaware that she had made this micromovement of the eyes. MH informed the client of the non-verbal response she made each time MH mentioned the word ‘wasp’. The situation was as though the phobia stimuli appeared each time the word ‘wasp’ was uttered by MH. The client acknowledged this. MH continued on the presumption that there was a wasp at the location of the fixed gaze and placed his hand into the location. The client displayed abject terror when MH moved the hand toward the client’s face and sighed with relief as the hand was moved away. The authors note a similar process can be witnessed in traumatized individuals where a hand moving toward the face can increase or intensify a fearful response. MH concluded that the client was reacting to (or perhaps re-enacting) an emotional memory that was associated with a specific location within the client’s peripersonal space. The eyes appeared to fixate on an ‘image,’ therefore MH described the ‘entity’ as an emotional memory image (EMI). This prompted MH to investigate other phobias with other clients and the same pattern was identified.

### Emotional memory image: Therapeutic utility

Thus, the hypothesis of EMIs coupling traumatic events to psychophysiological dis-ease arose from clinical observations grounded in psychotherapeutic theory, ultimately resulting in the proposal of our Split-second Unlearning (SSU) model and our Split-second Unlearning therapeutic approach (Hudson and Johnson, 2021).

The SSU model suggests that a person’s primitive brain engages a fear/defense cascade when faced with traumatic/adverse situations. This response is then learned by the body and an emotional memory image is created as an early warning detection system. This ensures that the person has extra time to avoid the situation in the future or to act in precisely the same way in order to survive. Split-second unlearning, therefore, interrupts this non-conscious fear response by interrupting the connection between the EMI and the body, within a split-second.

The coupling of emotional and mental imagery led to the development of psychodynamic therapeutic approaches that analyze the symbolic properties of mental images. Cognitive therapeutic approaches were developed incorporating mental imagery in order to adjust cognitive context and modify affective state. Often, therapeutic approaches use positive mental imagery to interrupt the interpretation of negative or ambiguous situations. This induces a positive mood (exposure to positive mental imagery) and desensitizes fearful situations manifested by mental imagery of fear-inducing objects or contexts (exposure to negative mental imagery). Once the suppressed information comes to the surface, an integration can take place, which has been proposed as a basis of mental health (Siegel, 2019).

Our Split-second Unlearning approach is fundamentally different to therapeutic approaches interacting with mental imagery at a *conscious* level. We argue in the Split-second Unlearning (SSU) model that the presence of the EMI is a marker for the non-conscious (repressed) trauma. We describe the process of revealing EMIs *via* micro-expressions associated with non-verbal communication. For example, a sharp peripheral peek in eye movement, lasting a split-second, or eye fixations of which the person is unaware, but which are observable by a therapist. The therapist then adopts approaches to interrupt and remove the EMI based on principles of unlearning, thereby uncoupling the emotional memory from the stress response as briefly outlined here.

The SSU process is educational. The client is encouraged to approach their presenting problem as a barrier to learning, where curiosity is utilized to embolden the client.

Curiosity enhances memory encoding by increasing the amount of attention and exploration of a person, which is the opposite of their fear driven stress response. The dopamine that is simultaneously released into the hippocampus encourages information seeking and enhances the consolidation of any acquired data (Gruber and Ranganath, 2019). The therapist then acts as an external source to feedback non-verbal communication, in order to interrupt, or clear the EMI. The therapist develops a rapport with the client, manifesting a state of curiosity for and between the client and therapist that sets the client at ease. Questions that illicit or cause the client to access their non-conscious process are used, i.e., “What would you like to work with today?” “How do you know when it is time to do your problem?”. The non-verbal response to this question yields the basis upon which the therapist begins the SSU model interaction. See Hudson and Johnson (2021) for further details of this approach.

### Emotional memory image: Refining the concept

A search of scholarly literature, including a systematic search of computerized research databases by MH and MIJ, failed to identify any instances of the prior use of the term ‘Emotional Memory Image.’ In 1899, however, Bernheim described the concept of ‘Sensitive Memory Image’ (SMI) in relation to his scholarly work on hypnosis (Bernheim, 2018). Bernheim suggested that whilst a person is in a hypnotic trance, their mind can adhere and respond to multimodal mental imagery. Bernheim wrote “You have a tickling sensation in your nose. The thought induced through hearing is reflected upon the center olfactory sensibility, where it awakens the sensitive memory - image of the nasal itching, as former impressions have created it and left it imprinted and latent. This memory sensation thus resuscitated, may be intense enough to cause the reflex act of sneezing … *In the same way the visual, acoustic and gustatory images succeed the suggested idea*” (Bernheim, 2018: 137–8).

Reflecting on Bernheim’s ideas about SMIs, we were struck by the term ‘sensitive’ because there may be instances when we do not experience an emotional response to a traumatic event. For example, if we accidentally burn ourselves as children, we do not necessarily experience an emotional response, but we do become ‘sensitive’ to it. This sensitivity may be physiological, in the form of peripheral and central sensitization to the injury, manifesting as a heightened pain experience that promotes fear-avoidance behavior on future encounters (i.e., psychological sensitivity). Nevertheless, we observed emotion to be central to individuals presenting under the broad spectrum of conditions associated with psychophysiological dis-ease.

Moreover, MH’s clients were not subject to hypnosis or therapeutic suggestion as to what the mental images should, could or ought to be. In fact, MH’s clients claimed to be unaware of the presence of mental images until their non-verbal communication was fed back to them. We postulated that the process of working with a client who is interacting with their presenting problem may create a momentary trance-like state and this ‘rapid-hypnosis’ could explain why the client is unaware (non-conscious) of their EMI and their non-verbal response to it. The client only appears to become more consciously aware of their EMI as they and the practitioner curiously interact with the EMI experience.

Thus, we postulated that EMIs could be mental images that are stored inside the mind, yet outside of a person’s conscious awareness, and formed as a result of an adverse experience, triggering an automatic stimulus response mechanism whenever anything resembling the original context occurs. Siegel proposes both an embodied and relational model: “*[the] mind extends beyond the boundaries of the skin, beyond a single skull and even a single body, to some kind of distributed process in which mind also arises from our social connections of energy and information flow shared among us.*” (Siegel, 2016) see also Sutton (2006) and Siegel (2019).

### Emotional memory image: Definition

Trauma induced, non-conscious, contiguously formed multimodal mental imagery, which triggers an amnesic, anachronistic, stress response within a split-second.

## Emotional memory image: Working definition

Previously, we described an emotional memory image as an “*[emotional memory] frozen in time and intrinsically linked to a set of physical reactions, including eye movement and/or fixation*” (Hudson and Johnson, 2021).

We refined our original definition by carefully considered terms defined in the American Psychological Association (APA) dictionary of terms and in other literature (Supplementary Material A—Table).

We chose the word ‘emotion’ to capture the experiential, behavioral, and physiological aspects of an individual’s attempt to cope with a significant personal event from an apparent or implied interaction with the world, and typically manifesting in a feeling. Emotion is integral in adverse traumatic events that result in a psychophysiological fight, flight, and freeze response. The fright response is central to our Split-second Unlearning model and is also clearly an emotional reaction, and therefore, we believe the word emotion to be appropriate.

We chose the word ‘memory’ to capture ‘retention’ over a period of time, and ‘retrieval’ or ‘reactivation’ of a representation of past experience, i.e., ‘longevity’ and ‘recurrence’ of a mental event. We considered the word ‘mental’ as an alternative to memory because ‘mental’ places the EMI within the domain of mind and not necessarily brain. The importance of ‘longevity’ and ‘recurrence’ seemed of critical relevance and best captured by the word ‘memory.’

The APA definition for emotional memory captures our stance: “*Memory for events that evoke an emotional response. Emotional memories can be either implicit (non-conscious) or explicit (conscious)*”.^1^

We chose the word ‘image’ to capture mental production and recollection of multimodal sensory information from experience or self-generated. We propose that the image is non-conscious as the client is unable to point to it and is oblivious to change in breathing, micro movements, micro expressions, pupil dilation, eye fixation or sharp peripheral peeks.

We have reflected further on the clarity and precision of our original definition to include the following aspects of the experience:

• ‘Imagined’ allowing the possibility of vicarious (imagined) trauma driving psychophysiological dis-ease;
• ‘Non-conscious’ to account for a cognitive process or event not explicitly in conscious experience or available to introspection or report. We rejected the alternative ‘preconscious,’ which concerns unattended mental contents that can be readily accessed and brought to awareness, because some clients never access and bring to conscious awareness the mental contents of the EMI;
• ‘Anachronistic’ to reflect the archaic nature of the emotional memory existing ‘out of its time in history’ (i.e., belonging to an earlier period). The physiological, biological, neurological, psychological, and behavioral response is always in the present, the *now*, whilst the EMI is an echo or an imprint from the past. The anachronistic (archaic) impact of the EMI on the body is from an entirely different time/space, even though the client may be physically in the exact same place. In other words, just because the client is in the ‘place’ does not mean that they are in the ‘space’;
• ‘Micro-expressions’ to reflect the physical manifestation of the EMI; and
• ‘Contiguous’ to reflect the traumatic impact of original spatio-temporal events in proximity, thus creating one-trial learning.

## Emotional memory image: Attributes

The definition of EMIs identifies the following key features:

1. The EMI is an emotional memory stored inside the mind.
2. The EMI is characterized by multimodal mental imagery.
3. The EMI is originally created as a result of an actual or imagined adverse experience (trauma induced).
4. The EMI is formed as part of a traumatic, spatio-temporal event, which triggers one-trial learning.
5. The EMI is triggered whenever the person is exposed to contexts similar to the original context(s) that created the EMI.
6. The EMI produces a ‘stress’ response within a split-second.
7. The EMI-triggered stress response is ‘outdated’ (anach ronistic).
8. The EMI is hidden within a deletion filter/mental amnesic state and outside of a person’s conscious awareness (non-conscious).
9. The EMI triggered stress response manifests most commonly as micro eye movements, eye fixation, pupil dilation and or sharp peripheral peeks.

We have refined our definitions and description of attributes of EMIs following appraisal of nomenclature and definitions from psychology. Table 1 provides a synopsis of the distinctive features of EMIs.

**TABLE 1:** **EMI Attributes**.

Attribute	Description	Explanation
Formed by an adverse experience	An adverse experience is a potentially traumatic event, and it may happen before 18 years of age [i.e., Adverse Childhood Experience (ACE)].	The EMI will present whenever anything similar to the original adverse experience happens at any time in life, e.g., a perceived imagined or real threat to life or autonomy. If there is conscious awareness of the original/source of the problem prior to consultation, then this precludes an EMI
Triggers a psychophysiological stress response	A psychophysiological stress response is the physiological or psychological response to internal or external stressors (stimuli)	When a person encounters a situation similar in context or content to the original situation the EMI is triggered and mediates a stress response. The psychophysiological stress response to the EMI can be any of the fear defense cascade freezing, flight or fight, tonic immobility, and quiescent immobility. Symptoms that may be associated with this stress include non-communicable diseases, acute/chronic pain, somatic tics, erratic behavior, chest pains, panic attacks, chronic fatigue, hyposensitivity hypersensitivity, selective mutism, stammering, catatonic limbs, irritable bowel syndrome
Comprises an emotional memory	An emotional memory is a memory for events that evoke an emotional response.	The non-conscious process created by the presence of the EMI can run automatically whenever similar contexts arise.
Characterized as mental imagery	Mental imagery is a quasi-perceptual experience that subjectively resembles the experience we have when we actually perceive something.	Mental imagery associated with the EMI may be of visual, auditory, olfactory, gustatory, tactile, and even proprioceptive in nature that can operate at conscious and subconscious levels
Outside of a person’s conscious awareness (non-conscious)	Non-conscious is an idea or impression that is present in the mind, but of which there is at the time no conscious knowledge or realization.	The EMI remains outside of the persons conscious awareness, so that it can respond to perceived threats ahead of time. A person accesses the EMI prior to talking about their problem. They will routinely access the EMI *via* micro expressions during the conversation. This action is non-conscious as the client is not consciously aware at the time that they are carrying out this action
EMIs are conveyed as micro-expressions during consultation	Micro-expressions are innate expressions lasting for a short moment resulting from emotional response. The person may convey a micro-expression repeatedly and may not realize they are doing so. Commonly, expressed with their face, hands, or voice.	These micro-expressions offer therapeutic utility and can present anywhere in the body but most commonly the face, eyes and asymmetrical body movements of the hands and arms. The micro-expressions may include a sharp peripheral peek or focal gaze avoiding a specific area within the peripersonal space whilst discussing the presenting problem or a sharp intake of breath or shift in breathing pattern
EMIs are formed as the result of a traumatic first-time experience, resulting in a contiguous learning loop.	Contiguity learning is a theory stating that if a pattern of stimulation and a response occur together in time and space, learning occurs by the formation of associations between them, so that the same stimulus pattern will elicit the same response on subsequent occasions.	The non-conscious mind adopts one-trial learning to survive any similar experiences in the future. The learnt response will automatically operate outside of the client’s awareness whenever they are within a context that is similar to the original. The action must be non-conscious and elicit the same response each time. If the client consciously recollects the first time that the event happened, then this will not directly necessitate the presence of an EMI. One trial learning with associated trauma compounds the space, time learning response.

We discuss each of the attributes of an EMI further below and offer a simple vignette to demonstrate these attributes in practice.

### Emotional memory image: Previous adverse experience

We propose that one-trial learning (Guthrie, 1930, 1935; Lachnit, 2003) of an adverse experience act as the original stimulus to form an EMI and that the EMI re-presents whenever a person encounters an adverse event in the future. However, we are offering a new line of enquiry where the person presents with psychophysiological dis-ease resulting from the lack of awareness of their original trauma.

For example, people presenting with arachnophobia have conscious knowledge of their fear of spiders. However, they are not conscious of the EMI which is the root cause of their problem. The EMI triggers the threat response system in any context that contains a spider (the stimulus). The stimulus is not the spider *per se*, which is directly within the current context of the person; rather, the stimulus is the stored EMI of a spider. The *threat* of the perceived spider dominates and is accessed *via* a split-second micro-movement often of the eye(s) that we interpret as a sharp peripheral peek. The sharp peripheral peek may be toward the EMI at the edge of the person’s peripersonal space, or alternatively it may be that the client avoids looking at a certain spot within their visual field; both denote the presence of an EMI. In the case of limited or zero eye movement (the Poker Face), this denotes that access to the EMI is being driven *via* a different modality, e.g., auditorily. If this is the case, then it is important to enquire of the client, “what can they hear?”, or “what are they telling themselves?” Otherwise, an attempt to clear the EMI can fail, as the therapist will be out of rapport with the client’s non-verbal communication. By bringing the existence, although not necessarily the content, of an EMI into the person’s conscious awareness, it is possible for a therapist to apply the SSU model to remove the EMI. This allows the person to run a number of mental checks, where they see themselves successfully dealing with the problem. Indeed, the therapist can check with the client at this point and discover that the client is running positive mental imagery to ‘test’ if the intervention has been successful or not. The client is consciously checking for any negative feelings that were previously associated with the presenting problem. The therapist can then glean validation of the success of the intervention by observing the client’s micro and macro expressions about the context. The client may also move their language from the present tense to the past, i.e., ‘I have a problem’ becomes ‘I had a problem.’ This non-conscious shift in tense can be pointed out by the therapist, which helps to give more validation to the client.

First-time experiences are more prevalent in infancy and early childhood. Therefore, an unprecedented experience such as burning one’s finger on a hot stove may create an EMI, which drives avoidance of fire. Thus, avoidance behavior can be viewed as a positive survival response driven by an initially negative EMI. This one-time learning does not require repetition for the child to remember that fire burns, it is a single lesson that lasts a lifetime. On the other hand, the initial benign investigation of a spider can result in arachnophobia. Prior to the entrance of the parent/caregiver to the scene, the child is curiously exploring the spider, a novel, unprecedented, first-time stimulus. Unlike fire, this new stimulus is not associated with a painful experience. The parent/caregiver’s reaction to the scene is abject terror, which alerts the child’s primitive system to a threat within the immediate vicinity. The only ‘new thing’ within this area is the spider and the child’s mind is updated by learning spider equates to threat. Therefore, the EMI is created *via* the transference of the parent/caregiver’s terror (fear) to the child who creates an EMI which negatively associates with the spider. Both experiences can be seen as preverbal traumas (for more insight to infant traumas see Coates, 2016 and Spiel et al., 2019), which receive a poor prognosis for young children in community based mental health (Finn et al., 2017). The Split-second Unlearning process, however, requires no verbal recollection of the original traumatic experience, seeking only to locate and clear the EMI (Hudson and Johnson, 2021).

### Emotional memory image: Psychophysiological ‘stress’ response

The APA defines ‘stress’ as “*the physiological or psychological response to internal or external stressors*,…*influencing how people feel and behave*”.^2^ We propose that the EMI is coupled to the innate set of evolutionary adapted stress responses (FFF) promoting fear and defense in the face of threatening situations such as predators and competitors. Assertion (fight) and evasion (flight) both serve as active arousal responses. Freezing can be an optimal survival strategy placing the organism into a temporarily static state when unable to escape or fight back as predators often detect and act according to movement of their prey. When all else fails, the last recourse may be collapsed immobility (Kozlowska et al., 2015). We argue that in modern settings EMIs can trigger freeze responses that, when held for years, can contribute to psychophysiological dis-ease, reducing ‘functional mobility.’ Thus, we predict that EMIs increase in size, proximity and intensity when triggered by external stimuli that are in some way similar to the original event causing the EMI. Under normal circumstances, after the danger has gone, the mending and recovery process may begin. We argue that individuals are not freed from the fear response until the EMI has been removed.

### Emotional memory image: Emotional memory

The APA describes emotional memories as “… *memor[ies] for events that evoke an emotional response. Emotional memories can be either implicit (non-conscious) or explicit (conscious)*” [see text footnote 1]. We propose that EMIs form implicit emotional memories that have been shown to manifest as conditioned freeze responses.

Functional neuroanatomical studies demonstrate that the affective system is embedded within cognitive and sensory systems, including brain regions associated with mental imagery. It has long been known that emotional states can be modified by mental images and that mental images can alter emotional states (Currie, 1995; Phillips, 2014). Emotional experiences emerge from distributed activity of various regions of the brain, including areas that subserve general functions (Lindquist and Barrett, 2008; Mackes et al., 2018). Subcortical regions of the limbic system are involved in processing implicit emotional memory (Ghaziri et al., 2018); the amygdala is associated with fear (Rosero et al., 2019; Zhang et al., 2020), the insula with pain and disgust (Corradi-Dell’Acqua et al., 2016; Riečanský and Lamm, 2019) and the orbitofrontal cortex with anger (Funayama et al., 2019). Hippocampal damage may result in the loss of residual emotional components associated with previous traumatic events (Gulyaeva, 2019).

### Emotional memory image: Mental image

Coupling of emotional memories and mental imagery is known to impact mood, cognition, and behavior in everyday life, especially when reliving the past or making plans for the future. The occurrence and nature of a broad spectrum of dysfunctional and maladaptive mental imagery can be a defining hallmark of a wide range of mental illnesses including obsessive compulsive disorder and dissociative disorders (Somer et al., 2021a). The COVID-19 pandemic has seen a marked rise of people from the post-millennial generation wishing to leave their day-to-day life in exchange for a reality shifting experience (Somer et al., 2021b). This need for an alternative reality may, at first appear to be a flight response as the individuals seek sanctuary within the mental images they can control, by exiting the current paradigm of mental images beyond their control. However, the compulsive need for dissociation points to a mass freeze response rather than flight or fight. People are unable to flee or fight pervasive threats within modern living such as inequality, social injustice, poor pay, government overreach into personal lives, and digital disinformation, misinformation and censorship. This creates disorientation and substantial mental threat to health and well-being. Lives are placed in ‘suspended animation,’ like a vulnerable animal afraid to move for fear it will alert a predator of its whereabouts. Unfortunately, for some people suicide is seen as the only means of escape from the stresses of modern living. In 2018, intentional self-harm (including suicide) was the third highest cause of death in adolescents in the United Kingdom (Office for National Statistics [ONS], 2022).

The APA defines ‘mental’ as “*referring to the mind or to processes of the mind*,… *it may refer to the cognitive processes involved in these events, to differentiate them from physiological processes*”^3^; an ‘image’ as “*a likeness or cognitive representation of an earlier sensory experience recalled without external stimulation”*^4^; and ‘mental imagery’ as “*cognitive generation of sensory input*… *recalled from experience or self-generated in a non-experienced form*”.^5^

We propose that mental imagery is central to an EMI and that this mental imagery presents as a phenomenal consciousness, involving information processed by the nervous system that is multimodal (e.g., visual, auditory, olfactory, gustatory, tactile, and proprioceptive). The EMI is self-generated in both experienced and non-experienced forms, and may or may not be ‘presented,’ i.e., non-conscious (Arditi et al., 1988; Lacey and Lawson, 2013; Spence and Deroy, 2013; Nanay, 2018). We consider mental imagery associated with EMIs to be present as a quasi-perceptual re-experience of a sensory event, stored inside the mind and similar to a stimulus (event) encountered in the past or a novel combination of stimuli. Often mental imagery is considered to be a re-experiencing of a quasi-perceptual auditory event, such as a voice or a musical tune. We propose that EMIs remain a phenomenal consciousness that continues to be ‘hidden’ from the individual, i.e., non-conscious.

In the classical view, mental imagery was considered to be consciously experienced. We align with more contemporary view, that conscious experience is not a necessary prerequisite for mental imagery and that mental imagery can operate at a non-conscious level. For example, people with blindsight report being blind yet are able to perform motor actions and behaviors appropriate to stimuli, such as moving a hand toward a thrown ball (Kentridge et al., 1999; Kouider and Dehaene, 2007). People with aphantasia report being incapable of mental imagery although at least some of these individuals experience unconscious mental imagery, i.e., mental imagery of which they are unaware (Luke, 2018; Koenig-Robert and Pearson, 2019; Nanay, 2021, see also Church, 2008; Emmanouil and Ro, 2014; Phillips, 2014; Brogaard and Gatzia, 2017), on unconscious mental imagery. Experimental research using forced choice paradigms following the presentation of subliminal stimuli demonstrate that perception can occur below the level of awareness (i.e., non-conscious perception). Although, Pearson et al. (2015) have described mental imagery as “the representation of sensory information, they do not point to mental imagery as being conscious or unconscious (see Nanay, 2021 for a fuller discussion on conscious or unconscious mental representations and mental imagery). We propose that EMIs exist as non-conscious entities.

### Emotional memory image: Non-conscious entity

The APA define non-conscious as *“describing that which is not explicitly in the contents of conscious experience*… *describing any cognitive process or event that is not available to introspection or report*”.^6^ In normal circumstances, cognitive processes such as future event forecasting, episodic memory, visual working memory and dreaming rely on mental imagery that is accessible to an individual in the form of conscious mental imagery, e.g., as visual mental imagery. Mental imagery is a vital precursor to guide successful planning and decision-making by facilitating a process of re-experiencing the past to model hypothetical futures. These forms of mental imagery are within the awareness of the client. Mental imagery is commonly heightened in diseases and may manifest as hallucinations, perceptions in the absence of an external stimulus that has the qualities of a real perception (Maróthi and Kéri, 2018; Díaz-Santos et al., 2021).

We propose that EMIs are beyond the conscious awareness of the client and are not mental images or imagery that a person can imagine or create at will (i.e., they are non-conscious). We propose that this simple rule of thumb distinguishes EMIs from other descriptions of mental imagery or synonyms that may be derived from the term: Emotional Memory Image[s]. Therefore, situations where the person has an awareness or can give any insight as to the nature of their mental images, are excluded from being EMIs.

In other words, individuals may be aware of a sensory disturbance, but unaware that an EMI is contributing to this disturbance. For example, individuals experiencing a sensory phenomenon such as phantom limb pain or body dysmorphia, are not aware that an EMI is contributing to (‘distorting’) their sensory experience. Put simply, they believe the room is dark (sensory dis-ease such as pain or body dysmorphia), but they are unaware that they are wearing sunglasses (the EMI). Nevertheless, we propose that although EMIs are not ‘visible’ to a person within their conscious perception, non-verbal communication will be present in the form of micro-gestures coming from the person at the point of EMI access.

### Emotional memory image: Formation, storage, and the deletion filter

Mental imagery is a top-down process, with the association cortices of various senses having a role in formation and storage. For example, visual mental imagery is produced and stored *via* processing in the association visual cortex, with the primary visual cortical areas having less of a role (Pearson et al., 2015). The reflex response to a visual threat (e.g., such as snakes and spiders) involves subcortical regions coupled to regions involved in emotional response, and regions involved in memory and cognition that influence the context of a mental image. Visual mental imagery is stored within the visuospatial sketchpad, divided into the visual cache, processing information about color, form, and identity. The inner scribe, processes information about spatial location and movement.

The visuospatial sketchpad has a critical role in the re-creation of images of real time or past events, enabling a person to ‘hold’ an image in the ‘mind’s eye.’ Indeed, trauma specialists have highlighted that unlike ordinary narrative memory, which changes and fades overtime, traumatic memory maintains its original strength (Van der Kolk and van der Hart, 1991) and can be described as ‘indelible’ (LeDoux, 1992). We propose that EMIs may continue to ‘cast a shadow over the unlived portions of a person’s life,’ preventing them from moving forward by driving feelings of inadequacy, or of being stuck or trapped.

In cases where a person has been subjected to a traumatic/adverse experience, full or partial neuropathological amnesia may occur (Van der Kolk et al., 2001; Nijenhuis et al., 2004; Casey et al., 2018). This type of dissociative survival mechanism is common in cases of sexual abuse and extreme violence (Van der Kolk and Fischer, 1995; Van der Kolk et al., 2001), with evidence suggesting that approximately 40% of people experience total amnesia and approximately 60% experience partial amnesia (Briere and Conte, 1993; Williams, 1994; IVSEA, 2015).

### Amnesia and the deletion filter

The phenomenon of amnesia was first explored in the nineteenth century (Janet, 1889; Van der Kolk et al., 1989). The onset of World War I (Myers, 1915; Thom and Fenton, 1920) provided the backdrop against which soldiers who suffered trauma in combat were examined and documented in great detail within the psychiatric literature. Indeed, dissociative traumatic amnesia has been identified within the criteria for Post-Traumatic Stress Disorder (DSM-5 American Psychiatric Association [APA], 2013). For an overview of amnesia after trauma see Salmona (2018). We propose that EMIs were created as a mental depiction of what transpired during a traumatic/adverse event and that their inaccessibility is part of a human’s adaptative ‘freeze’ response.

We have explored this aspect of the non-conscious, amnesic nature of EMIs by considering the possibility of a ‘deletion filter,’ as described in the psychotherapeutic model of Neuro-Linguistic Programming (NLP). NLP is an eclectic psychotherapeutic model (Bandler and Grinder, 1975) that uses the meta-model and meta-questioning to delve into areas of communication that the client fails to acknowledge at first. The client initially generalizes, distorts, and deletes information about their experience and then generalizes, distorts and deletes the language that they choose to describe their experience. Moreover, the distortion process can influence a person’s self-image when looking out at the world and conveying their narrative of self. This may distort how the person sees themselves. Therefore, the spoken word is in effect a map of a map (O’Connor and McDermott, 1996), that is, two steps removed from the experience of the senses (Knight, 2012). If the client on some level is non-consciously deleting incoming data, that challenges or threatens the *status quo*, in order to maintain the survival strategy, then perhaps the EMI is a key part of the deletion process.

In the example of a spider phobia, the deletion, distortion and generalization filters are as follows:

• *Deletion:* The human subject presents with a phobia of spiders without conscious knowledge of the original experience.
• *Distortion:* The human subject perceives spiders to be a terrifying threat to their survival. The human subject has a synesthetic response to the stimulus (real or imagined), culminating in a feeling of panic or terror.
• *Generalization:* The survival response mechanism perceives that all spiders are a threat to their survival and automatically triggers a threat response whenever the stimulus (imagined or real) is nearby.

Thus, we argue that traumatic amnesia, “[the] *inability to recall key features of the trauma (usually dissociative amnesia; not due to head injury, alcohol, or drugs)*” (DSM-5 American Psychiatric Association [APA], 2013), prevents the person from recognizing that an EMI is the source of their psychophysiological dis-ease, i.e., the person may not present with conscious knowledge of having experienced a severe trauma. Approaches to repair the broken psyche of the detached personality (Nijenhuis et al., 2010) or the amnesic state associated with sexual abuse and violence, for example, are well documented (Van der Kolk et al., 2001; Ecker, 2015, 2018 for review). Classically, the person is encouraged to revisit the traumatic experience and integrate new learning about the original event (Van der Kolk et al., 1989). This drives higher brain functions to exert control over structures mediating fear, such as the amygdala. This downgrades the freeze response, allowing for a more autobiographical memory (Nijenhuis et al., 2004; Ecker, 2018). Our Split-second Unlearning approach is fundamentally different.

### Emotional memory image: Micro-expressions

The APA define expression as an “*external manifestation of an internal condition or characteristic. The term, however, is most often used in reference to the communication of a thought, behavior, or emotion, as in emotional expression or facial expression*”.^7^ Svetieva and Frank (2016) define a micro-expression as expressions that lasts for a short moment that are innate and result from voluntary and involuntary emotional responses occurring simultaneously and conflicting with one another. They suggest that this occurs when a person tries to conceal their innate emotional response mediated via the amygdala causing and manifesting as a brief display of true emotion followed by a false emotional reaction.

We propose that EMIs are intrinsically linked to non-verbal, non-conscious, momentary expressions when a person ‘accesses’ their EMI. Common micro-expressions are:

• Sharp peripheral peeks which will focus on the same exact spot as the client chats about their presenting problem.
• Change in facial color or skin tone.
• Sharp intake of breath.
• Physiological regression—the client’s face looks like that of a vulnerable child.
• Asymmetry when talking about the presenting problem.
• Pupil dilation.
• Eye fixation.
• Eye avoidance—the eyes look everywhere except at the EMI.

We chose to describe these involuntary, non-verbal, and subtle external manifestations as micro-expressions, in line with descriptions by Ekman and Friesen (1969) of non-conscious facial expressions of up to 0.5 s in duration. Micro-expressions momentarily expose the emotional state of a person, resulting from conscious suppression or unconscious repression (Zhang et al., 2022). Such micro-expressions are often misinterpreted or missed altogether. We propose that failure to manifest a micro-expression such as a sharp peripheral peek, may be suggestive of a non-visual component to the EMI, e.g., an auditory EMI. Alternatively, a client’s avoidance of a specific spot within the visual field without a corresponding micro-expression, may be indicative of a large EMI being present. This is outside of the client’s conscious awareness and the client is unknowingly afraid to access it.

### Attributes: Summary

A schematic representation of the relationship of the EMI to psychophysiological dis-ease and dysregulation of the HPA axis is provided in Supplementary Material B—Figure.

Our discussion of the attributes of EMIs enables us to distinguish the EMI from a variety of synonyms used by computerized databases as subject topics and terms. This includes emotional memory, mental image(ry), mental representation, mental visualizations, mental picture, imagination image, thought-image, impression, auditory image, hallucinations. Table 2 provides a synopsis of the features that distinguish EMI from similar phenomena.

**TABLE 2:** EMI uniqueness in relation to similar phenomenon: What they are and what they are not.

Phenomenon	Formed by an adverse experience	Triggers a psychophys-iological ‘stress’ response	Comprises an emotional memory	Characterized as mental imagery	Outside of a person’s conscious awareness (non-conscious)	Presence of (or interaction with) EMI conveyed as micro-expressions during consultation
EMI	Always	Always	Always	Always	Always	Always
Emotional Memory	Always	Always	Always	Always	Sometimes	Always
Mental Imagery	Sometimes	Sometimes	Sometimes	Always	Sometimes	Sometimes
Sensitive Memory Image (Bernheim)	Always	Always	Always	Always	Sometimes	Always
Mental images	Sometimes	Sometimes	Sometimes	Sometimes	Sometimes	Sometimes
Picturing	Sometimes	Sometimes	Sometimes	Sometimes	Never	Sometimes
Hallucinations	Sometimes	Sometimes	Sometimes	Sometimes	Never	Sometimes
Mental visualizations	Sometimes	Sometimes	Sometimes	Sometimes	Never	Never
Visualization	Sometimes	Sometimes	Sometimes	Sometimes	Never	Never
Mental picture	Sometimes	Sometimes	Sometimes	Sometimes	Never	Never
Imagination image	Sometimes	Sometimes	Sometimes	Sometimes	Never	Never
Mental representation	Sometimes	Sometimes	Sometimes	Sometimes	Never	Sometimes
Thought-image	Sometimes	Sometimes	Sometimes	Sometimes	Never	Sometimes
Impression	Sometimes	Sometimes	Sometimes	Sometimes	Never	Never
Auditory image	Sometimes	Sometimes	Sometimes	Sometimes	Sometimes	Sometimes

## Vignette: The child who was terrified to go to bed

This clinical vignette demonstrates the attributes of the EMI.

A 12-year-old female had been attending Child and Adolescent Mental Health Services (CAMHS) for 7 years. Her presenting problem was a morbid fear of going to bed in her own bedroom. The client was 3 years old when the family had moved house in October of that year. MH deduced that the family had moved home later in the year (when there were darker nights) and that the child’s new bedroom was darker than her original. The mother also shared that she sensed there was something between her daughter and the bedroom, so had redecorated it several times, switched rooms around and even shared the bedroom with her other daughter, to no avail.

This first-time move of house created an EMI which recurred each day causing great distress to the child and her family. MH worked with the child for 30 min and cleared an EMI that was created when the family had moved to a new house. The following day mum telephoned and reported the child had simply gone to bed as if there had never been a problem. A 1 and 6 months follow up call confirmed that the child had not experienced any further problems.

### Application of emotional memory image definition within the vignette

• *Trauma induced* – Dark bedroom.
• *Non-conscious* – The mother told of the house move, the child had no conscious memory it.
• *Contiguously formed* – The fear was created on the very first night at the new house.
• *Multimodal mental imagery* – Visual mental image/representation of the dark bedroom.
• *Triggers* – Each night the stress response would ‘fire-up’ at bedtime.
• *Amnesic* – The child’s awareness of the EMI and prior event were non-conscious.
• *Anachronistic* – The EMI was formed 7 years earlier yet ‘fired’ each night in real time.
• *Stress response* – The child was thrown into inconsolable abject terror each bedtime.
• *Within a split-second* – Mother reported her child’s fear began instantly at the new house.

## Conclusion

The human experience of life remains as illusive and magical as it has for millennia. Science has taken great strides in many different directions, yet the mind still throws many questions into the mix. Eye Movement Desensitization and Reprocessing (EMDR) and talking therapies that can help a person acknowledge their problems, let go of the emotion and take onboard new learning, are showing that an interaction with the mind can produce changes in the body. The EMI therefore, stands at the gateway to a whole new area of research and development into resolving childhood preverbal trauma. Given that the pandemic of 2020 was presented to the public as an unprecedented event, the potential for EMI’s being created in the majority of the population is vast. Low-cost treatment methods that are fast and scalable must be given priority to run alongside the more expensive historical approaches. Exploration of the concept of EMIs offers opportunities to shed light on the rise of psychophysiological dis-ease and to show many disorders as natural orders ergo responses from the autonomic nervous system to an ever-present threat, an EMI. We hope this article catalyzes further scholarship and research.

## Author contributions

Both authors contributed equally to the conception and writing of the review and approved the submitted version.

## Footnotes

1
https://dictionary.apa.org/emotional-memory

2
https://dictionary.apa.org/stress/

3
https://dictionary.apa.org/mental

4
https://dictionary.apa.org/image

5
https://dictionary.apa.org/mental-imagery

6
https://dictionary.apa.org/non-conscious

7
https://dictionary.apa.org/expression

## References

[B1] American Psychiatric Association [APA] (2013). *Diagnostic and Statistical Manual of Mental Disorders*, 5th Edn. Virginia: American Psychiatric Association. 10.1176/appi.books.9780890425596

[B2] Arditi A. J. Holtzman D. Kosslyn S. M. (1988). Mental imagery and sensory experience in congenital blindness. *Neuropsychologia* 26 1–12. 10.1016/0028-3932(88)90026-7 3362335

[B3] Bandler R. Grinder J. (1975). *The Structure of Magic*, Vol. 1. Santa Clara, CA: Palo Alto.

[B4] Bernheim H. (2018). *Suggestive Therapeutics: A Treatise on the Nature and Uses of Hypnotism.* Berlin: Franklin Classics.

[B5] Briere J. Conte J. (1993). Self-reported amnesia in adults molested as children. *J. Trauma. Stress* 6 21–31. 10.1002/jts.2490060104

[B6] Brogaard B. Gatzia D. E. (2017). Unconscious imagination and the mental imagery debate. *Front. Psychol.* 8:799. 10.3389/fpsyg.2017.00799 28588527 PMC5440590

[B7] Casey P. R. Strain J. J. Trans. Crocq M. A. Boehrer A. (2018). *Les Troubles Lieìs Aux Traumatismes et Aux Facteurs de Stress*, (Paris: Elsevier Masson) Google Scholar.

[B8] Church J. (2008). The hidden image: A defense of unconscious imagining and its importance. *Am. Imago* 65 379–404. 10.1353/aim.0.0024 34409987

[B9] Coates S. W. (2016). Can babies remember trauma? symbolic forms of representation in traumatized infants. *J. Am. Psychoanal. Assoc.* 64 751–776. 10.1177/0003065116659443 27474085

[B10] Corradi-Dell’Acqua C. Tusche A. Vuilleumier P. Singer T. (2016). Cross-modal representations of first-hand and vicarious pain, disgust and fairness in insular and cingulate cortex. *Nat. Commun.* 7 10904–10904. 10.1038/ncomms10904 26988654 PMC4802033

[B11] Currie G. (1995). Visual imagery as the simulation of vision. *Mind Lang* 10 25–44. 10.1111/j.1468-0017.1995.tb00004.x

[B12] Díaz-Santos M. Monge Z. A. Salazar R. D. Gilmore G. C. Neargarder S. Cronin-Golomb A. (2021). Increasing contrast improves object perception in Parkinson’s Disease with visual hallucinations. *Mov. Disord. Clin. Practice* 8 51–59. 10.1002/mdc3.13104 33426159 PMC7780942

[B13] Ecker B. (2015). Memory reconsolidation understood and misunder-stood. *Int. J. Neuropsychother.* 3 2–46. 10.12744/ijnpt.2015.0002-0046

[B14] Ecker B. (2018). Clinical translation of memory reconsolidation research: Therapeutic methodology for transformational change by erasing implicit emotional learnings driving symptom production. *Int. J. Neuropsychother.* 6 1–92. 10.12744/ijnpt.2018.0001-0092

[B15] Ekman P. Friesen W. V. (1969). Nonverbal leakage and clues to deception. *Psychiatry* 32 88–106. 10.1080/00332747.1969.11023575 5779090

[B16] Emmanouil T. A. Ro T. (2014). Amodal completion of unconsciously presented objects. *Psychon. Bull. Rev.* 21 1188–1194. 10.3758/s13423-014-0590-9 24671775

[B17] Finn H. Warner E. Price M. Spinazzola J. (2017). The boy who was hit in the face: somatic regulation and processing of preverbal complex trauma. *J. Child Adolescent Trauma* 11 277–288. 10.1007/s40653-017-0165-9 32318157 PMC7163863

[B18] Funayama M. Koreki A. Muramatsu T. Mimura M. Kato M. Abe T. (2019). Impairment in judgement of the moral emotion guilt following orbitofrontal cortex damage. *J. Neuropsychol.* 13 550–563. 10.1111/jnp.12158 29673084

[B19] Ghaziri J. Tucholka A. Girard G. Boucher O. Houde J.-C. Descoteaux M. (2018). Subcortical structural connectivity of insular subregions. *Sci. Rep.* 8 8596–8596. 10.1038/s41598-018-26995-0 29872212 PMC5988839

[B20] Gruber M. J. Ranganath C. (2019). How curiosity enhances hippocampus-dependent memory: The prediction, appraisal, curiosity, and exploration (PACE) framework. *Trends Cogn. Sci*. 23, 1014–1025. 10.1016/j.tics.2019.10.003 31706791 PMC6891259

[B21] Gulyaeva N. V. (2019). Functional neurochemistry of the ventral and dorsal hippocampus: Stress, depression, dementia and remote hippocampal damage. *Neurochem. Res.* 44 1306–1322. 10.1007/s11064-018-2662-0 30357653

[B22] Guthrie E. R. (1930). Conditioning as a principle of learning. *Psychol. Rev.* 37 412–428. 10.1037/h0072172

[B23] Guthrie E. R. (1935). *The Psychology of Learning.* New York, NY: Harper & Row.

[B24] Hudson M. Johnson M. I. (2021). Split-second Unlearning: Developing a theory of psychophysiological dis-ease. *Front. Psychol.* 12:716535. 10.3389/fpsyg.2021.716535 34912263 PMC8666476

[B25] IVSEA,. (2015). *2015-IVSEA Survey.* Available online at: https://www.memoiretraumatique.org/campagnes-et-colloques/2015-campagne-stop-au-deni.html (accessed October 11, 2022).

[B26] Janet P. (1889). *L’automatisme Mental.* Paris: Alcan.

[B27] Kentridge R. W. Heywood C. A. Weiskrantz L. (1999). Attention without awareness in blindsight.Proc. *R. Soc. Lond. B* 266 1805–1811. 10.1098/rspb.1999.0850 10518327 PMC1690198

[B28] Knight J. (2012). Deletion, distortion and data collection: The application of the neuro-linguistic progamming (nlp) meta-model in qualitative interviews. *Australas. J. Market Soc. Res.* 20 15–21.

[B29] Koenig-Robert R. Pearson J. (2019). Decoding the contents and strength of imagery before volitional engagement. *Sci. Rep.* 9:3504. 10.1038/s41598-019-39813-y 30837493 PMC6401098

[B30] Kouider S. Dehaene S. (2007). Levels of processing during non-conscious perception: A critical review of visual masking. *Phil. Trans. R. Soc. B* 362 857–875. 10.1098/rstb.2007.2093 17403642 PMC2430002

[B31] Kozlowska K. Walker P. McLean L. Carrive P. (2015). Fear and the defense cascade: clinical implications and management. *Harvard Rev. Psychiatry* 23 263–287. 10.1097/HRP.0000000000000065 26062169 PMC4495877

[B32] Lacey S. Lawson R. (eds) (2013). *Multisensory Imagery.* New York, NY: Springer. 10.1007/978-1-4614-5879-1

[B33] Lachnit H. (2003). “The principle of contiguity,” in *Principles of Learning and Memory*, eds Kluwe R. H. Lüer G. Rösler F. (Basel: Birkhäuser), 10.1007/978-3-0348-8030-5_1

[B34] LeDoux J. E. (1992). “Emotion as memory: Anatomical systems underlying indelible neural traces,” in *The Handbook of Emotion and Memory*, ed. Christianson S.-A. (Hillsdale, NJ: Lawrence Erlbaum Associates), 289–297.

[B35] Lindquist K. A. Barrett L. F. (2008). Constructing emotion: The experience of fear as a conceptual act. *Psychol. Sci.* 19 898–903. 10.1111/j.1467-9280.2008.02174.x 18947355 PMC2758776

[B36] Luke D. (2018). Reply to “Ayahuasca turned on my mind’s eye”: A case of acquired versus congenital aphantasia, as evidenced with DMT use? *J. Psychedelic Stud.* 2 97–98. 10.1556/2054.2018.014

[B37] Mackes N. K. Golm D. O’Daly O. G. Sarkar S. Sonuga-Barke E. J. S. Fairchild G. (2018). Tracking emotions in the brain – revisiting the empathic accuracy task. *Neuroimage* 178 677–686. 10.1016/j.neuroimage.2018.05.080 29890323 PMC6057276

[B38] Maróthi R. Kéri S. (2018). Enhanced mental imagery and intact perceptual organization in schizotypal personality disorder. *Psychiatry Res.* 259 433–438. 10.1016/j.psychres.2017.11.015 29131991

[B39] Myers C. S. (1915). A contribution to the study of shell-shock. *Lancet* 185 316–320. 10.1016/S0140-6736(00)52916-X

[B40] Nanay B. (2018). Multimodal mental imagery. *Cortex* 105 125–134. 10.1016/j.cortex.2017.07.006 28801065 PMC6079145

[B41] Nanay B. (2021). Unconscious mental imagery. *Phil. Trans. R. Soc. B* 376:20190689. 10.1098/rstb.2019.0689 33308067 PMC7741084

[B42] Nijenhuis E. R. S. van der Hart O. Kruger K. Steele K. (2004). Somatoform dissociation, reported abuse and animal defence-like reactions. *Aust. N. Z. J. Psychiatry* 38, 678–686. 10.1080/j.1440-1614.2004.01441.x 15324331

[B43] Nijenhuis E. van der Hart O. Steele K. (2010). Trauma-related structural dissociation of the personality. *Act. Nerv. Super.* 52 1–23. 10.1007/BF03379560

[B46] O’Connor J. McDermott I. I (1996). *Principles of NLP.* San Francisco, CA: Harper Collins.

[B47] Office for National Statistics [ONS] (2022). *Deaths Registered in England and Wales: 2021.* Available online at: https://www.ons.gov.uk/peoplepopulationandcommunity/birthsdeathsandmarriages/deaths/bulletins/deathsregistrationsummarytables/latest (accessed October 12, 2022).

[B48] Pearson J. Naselaris T. Holmes E. A. Kosslyn S. M. (2015). Mental imagery: Functional mechanisms and clinical applications. *Trends Cogn. Sci.* 19 590–602. 10.1016/j.tics.2015.08.003 26412097 PMC4595480

[B49] Phillips I. (2014). “Lack of imagination: Individual differences in mental imagery and the significance of consciousness,” in *New Waves in Philosophy of Mind*, eds Kallestrup J. Sprevak M. (London: Palgrave Macmillan), 278–300. 10.1057/9781137286734_14

[B50] Riečanský I. Lamm C. (2019). The role of sensorimotor processes in pain empathy. *Brain Topogr.* 32 965–976. 10.1007/s10548-019-00738-4 31705422 PMC6882755

[B51] Rosero M. A. Winkelmann T. Pohlack S. Cavalli J. Nees F. Flor H. (2019). Memory-guided attention: Bilateral hippocampal volume positively predicts implicit contextual learning. *Brain Struct. Funct.* 224 1999–2008. 10.1007/s00429-019-01887-9 31104120

[B52] Salmona M. (2018). Traumatic memory: Sexual abuse and psychological trauma. *Les Cahiers de la Justice* 1 69–87. 10.3917/cdlj.1801.0069 18052372

[B53] Siegel D. J. (2016). *Mind: A Journey to the Heart of Being Human.* New York, NY: WW Norton & Company.

[B54] Siegel D. J. (2019). The mind in psychotherapy: An interpersonal neurobiology framework for understanding and cultivating mental health. *Psychol. Psychother.* 92 224–237. 10.1111/papt.12228 31001926

[B55] Somer E. Abu-Rayya H. M. Brenner R. (2021a). Childhood trauma and maladaptive daydreaming: Fantasy functions and themes in a multi-country sample. *J. Trauma Dissociation* 22 288–303. 10.1080/15299732.2020.1809599 32845809

[B56] Somer E. Carde?a E. Catelan R. F. Soffer-Dudek N. (2021b). Reality shifting: Psychological features of an emergent online daydreaming culture. *Curr. Psychol.* [Epub ahead of print]. 10.1007/s12144-021-02439-3 34744401 PMC8556810

[B57] Spence C. Deroy O. (2013). “Crossmodal imagery,” in *Multisensory Imagery*, eds Lacey S. Lawson R. (New York, NY: Springer), 157–183. 10.1007/978-1-4614-5879-1_9

[B58] Spiel S. Lombardi K. L. DeRubeis-Byrne L. (2019). Treating traumatized children: Somatic memories and play therapy. *J. Infant Child Adolesc. Psychother.* 18 1–12. 10.1080/15289168.2019.1566974

[B59] Sutton J. (2006). Introduction: Memory, embodied cognition, and the extended mind. *Philosophical. Psychol.* 19 281–289. 10.1080/09515080600702550

[B60] Svetieva E. Frank M. G. (2016). Empathy, emotion dysregulation, and enhanced microexpression recognition ability. *Motiv. Emot.* 40 309–320. 10.1007/s11031-015-9528-4

[B61] Thom D. A. Fenton N. (1920). Amnesias in war cases. *Am. J. Insanity* 76 437–448. 10.1176/ajp.76.4.437

[B62] Van der Kolk B. A. Brown P. van der Hart O. (1989). Pierre Janet on post-traumatic stress. *J. Traumatic Stress* 2 365–378. 10.1002/jts.2490020403

[B63] Van der Kolk B. A. Fischer R. (1995). Dissociation and the fragmentary nature of traumatic memories: Overview and exploratory study. *J. Traumatic Stress* 8 505–536. 10.1002/jts.2490080402 8564271

[B64] Van der Kolk B. A. Hopper J. W. Osterman J. E. (2001). Exploring the nature of traumatic memory: Combining clinical knowledge with laboratory methods. *J. Aggress. Maltreat. Trauma* 4 9–31. 10.1300/J146v04n02_02

[B65] Van der Kolk B. A. van der Hart O. (1991). The intrusive past: The flexibility of memory and the engraving of trauma. *Am. Imago* 48 425–454.

[B66] Williams L. M. (1994). Recall of childhood trauma: A prospective study of women’s memory of child sexual abuse. *J. Consult. Clin. Psychol.* 62 1167–1176. 10.1037/0022-006X.62.6.1167 7860814

[B67] Zhang T. Zong Y. Zheng W. Chen C. L. P. Hong X. Tang C. (2022). Cross-database micro-expression recognition: A benchmark. *IEEE Trans. Knowl. Data Eng.* 34 544–559. 10.1109/TKDE.2020.2985365

[B68] Zhang Y. Chen S. Deng Z. Yang J. Yuan J. (2020). Benefits of implicit regulation of instructed fear: Evidence from neuroimaging and functional connectivity. *Front. Neurosci.* 14:201. 10.3389/fnins.2020.00201 32231516 PMC7082334

