# Supplementary material for: Definition and attributes of the emotional memory images underlying psychophysiological dis-ease
Source: Front Psychol. 2022 Nov 14;13:947952. doi: 10.3389/fpsyg.2022.947952 (PMC9702567; doi:10.3389/fpsyg.2022.947952)

# Supplemental Material A - Table. Nomenclature

APA = American Psychological Association

| **Term** | **Definition** | **Source(s)** |
| --- | --- | --- |
| Emotion | n.  a complex reaction pattern, involving experiential, behavioural, and physiological elements, by which an individual attempts to deal with a personally significant matter or event. The specific quality of the emotion (e.g., fear, shame) is determined by the specific significance of the event. For example, if the significance involves threat, fear is likely to be generated; if the significance involves disapproval from another, shame is likely to be generated. Emotion typically involves feeling but differs from feeling in having an overt or implicit engagement with the world. —emotional adj. | American Psychological Association  <https://dictionary.apa.org/emotion> |
| Memory | n.  1. the ability to retain information or a representation of past experience, based on the mental processes of learning or encoding, retention across some interval of time, and retrieval or reactivation of the memory.  2. specific information or a specific past experience that is recalled.  3. the hypothesized part of the brain where traces of information and past experiences are stored. See memory storage, memory system. See also explicit memory; immediate memory; implicit memory; long-term memory; short-term memory. | American Psychological Association  <https://dictionary.apa.org/memory> |
| Imagery | **n.**  1. cognitive generation of sensory input from the five senses, individually or collectively, which is recalled from experience or self-generated in a nonexperienced form.  2. mental images considered collectively, or the particular type of imagery characteristic of an individual, such as visual imagery. See imagery cue; imagery training; kinaesthetic imagery. | American Psychological Association  <https://dictionary.apa.org/imagery> |
| Mental | adj.  1. of or referring to the mind or to processes of the mind, such as thinking, feeling, sensing, and the like.  2. phenomenal or consciously experienced. In contrast to physiological or physical, which refer to objective events or processes, mental denotes events known only privately and subjectively; it may refer to the cognitive processes involved in these events, to differentiate them from physiological processes. | American Psychological Association  <https://dictionary.apa.org/mental> |
| Emotional memory | Memory for events that evoke an emotional response. Emotional memories can be either implicit (nonconscious) or explicit (conscious). In laboratory studies with nonhuman animals, implicit emotional memory is demonstrated through such phenomena as conditioned fear (see [avoidance conditioning](about:blank)) and [freezing behavior](about:blank); additional paradigms exist to study implicit emotional memory in humans. Explicit emotional memory is manifested when individuals reexperience the original emotions engendered by an event (e.g., terror when describing an accident, joy when describing a close family member’s wedding). Functional neuroanatomy suggests that the encoding process for implicit emotional memories centers around the [amygdala](about:blank) in the subcortical portions of the limbic system, and [adrenergic](about:blank) and [dopaminergic](about:blank) mechanisms in particular. Indeed, a person whose amygdala is damaged may calmly recall the details of a traumatic event without showing any residual fear in relation to that event. In contrast, encoding of explicit emotional memories involves the cortical regions of the limbic system, such as the [hippocampus](about:blank). Emotional memory is distinct from the more general phenomenon of enhanced storage and retrieval of emotional stimuli, which for example is seen when experimental participants recall aversive nouns from a word list better than they recall neutral items. Also called affective memory. | American Psychological Association  [https://dictionary.apa.org/emotional-memory](about:blank) |
|  | The ability to consciously remember aspects of those experiences; in other words, the term is used to describe the effects of emotion on [episodic memory](about:blank). | [Encyclopedia of the Sciences of Learning](about:blank)  [https://link.springer.com/referenceworkentry/10.1007%2F978-1-4419-1428-6_1008](about:blank) |
| Emotional image(ry) | APA – no entry |  |
| Emotional mental | APA – no entry |  |
| Memory image(ry) | APA - no entry |  |
| Memory trace | a hypothetical modification of the nervous system that encodes a representation of information or a learning experience. See [engram](about:blank) memory illusion | American Psychological Association  <https://dictionary.apa.org/memory-trace> |
| Memory illusion | a distortion in remembering, analogous to a perceptual illusion, in which one remembers inaccurately or remembers something that in fact did not occur. The Deese–Roediger–McDermott paradigm is a memory illusion. See [false memory](about:blank). | American Psychological Association  <https://dictionary.apa.org/memory-illusion> |
| Mental image(ry) | APA – no entry |  |
|  | The mental image or mental representation is defined as what an individual can feel, hear, see or taste in one’s own mind. However, the stimulus which creates the image is not actually seen, heard, felt, tasted, or smelt. | [The Elementary School Journal](about:blank)  [https://www.journals.uchicago.edu/doi/abs/10.1086/459733?journalCode=esj](about:blank) |
|  | A Quasi-perceptual experience, experience that subjectively resembles the experience we have when we actually perceive something | [Encyclopedia of Consciousness](about:blank)  <https://www.sciencedirect.com/topics/psychology/mental-imagery> |
| Memory mental | APA – no entry |  |
| Emotional memory image(ry) | APA – no entry |  |
| Emotional mental image(ry) | APA – no entry |  |
| Emotional memory mental image(ry) | APA – no entry |  |
| Nonconscious | adj.  1. describing that which is not explicitly in the contents of conscious experience.  2. describing any cognitive process or event that is not available to introspection or report.  3. a synonym for unconscious. Compare preconscious; subconscious. | American Psychological Association  <https://dictionary.apa.org/nonconscious> |
| Representation | n. that which stands for or signifies something else. For example, in cognitive psychology the term denotes a mental representation, whereas in psychoanalytic theory it refers to an introject (see introjection) of a significant figure or to a symbol for a repressed impulse. —represent vb. —representational adj. —representative adj. | American Psychological Association  [https://dictionary.apa.org/representation](about:blank) |
| Mental representation | A hypothetical entity that is presumed to stand for a perception, thought, memory, or the like during cognitive operations. | American Psychological Association  [https://dictionary.apa.org/mental-representation](about:blank) |
| Imagination | The faculty that produces ideas and images in the absence of direct sensory data, often by combining fragments of previous sensory experiences into new syntheses. | American Psychological Association  [https://dictionary.apa.org/imagination](about:blank) |
|  | The positing of an object as a nothingness. | Britannica - Psychology of Imagination  [https://www.britannica.com/topic/The-Psychology-of-Imagination](about:blank) |
| Visualization | The process of creating a visual image in one’s mind or mentally rehearsing a planned movement in order to learn skills or enhance performance. | American Psychological Association  [https://dictionary.apa.org/visualization](about:blank) |
| Hallucinations | A false sensory perception that has a compelling sense of reality despite the absence of an external stimulus. | American Psychological Association  [https://dictionary.apa.org/hallucination](about:blank) |
| Impression | One’s initial perception of another person, typically involving a positive or negative evaluation as well as a sense of physical and psychological characteristics. | American Psychological Association  [https://dictionary.apa.org/first-impression](about:blank) |
| Auditory image | APA – no entry |  |
|  | Auditory imagery is a complex process by which an individual generates and processes mental images in the absence of sound perception—“hearing with the mind's ear.” | Janata 2012  <https://www.academia.edu/28067566/Acuity_of_mental_representations_of_pitch?auto=citations&from=cover_page> |
| Thought-image | APA - no entry |  |
| Mental visualizations | APA - no entry |  |
| Mental picture | APA - no entry |  |
| Picturing | APA – no entry |  |
|  | Picture superiority effect  The tendency for a picture or drawing to be remembered better than the name of the pictured object. For example, people are more likely to remember “dog” if they see a drawing of a dog than if they see the word dog. | American Psychological Association  <https://dictionary.apa.org/picture-superiority-effect> |
| Sensitive memory image | APA – no entry |  |
| Stress | n.  1. the physiological or psychological response to internal or external stressors. Stress involves changes affecting nearly every system of the body, influencing how people feel and behave. For example, it may be manifested by palpitations, sweating, dry mouth, shortness of breath, fidgeting, accelerated speech, augmentation of negative emotions (if already being experienced), and longer duration of stress fatigue. Severe stress is manifested by the general adaptation syndrome. By causing these mind–body changes, stress contributes directly to psychological and physiological disorder and disease and affects mental and physical health, reducing quality of life. See also chronic stress. [first described in the context of psychology around 1940 by Hungarian-born Canadian endocrinologist Hans Selye (1907–1982)]  2. in linguistics, emphasis placed on a word or syllable in speech, generally by pronouncing it more loudly and deliberately than its neighbouring units and slightly prolonging its duration. See also accent. | American Psychological Association  [https://dictionary.apa.org/stress](about:blank) |
| Expression | n.  an external manifestation of an internal condition or characteristic … The term, however, is most often used in reference to the communication of a thought, behavior, or emotion, as in emotional expression or facial expression. | American Psychological Association  [https://dictionary.apa.org/expression](about:blank) |
| Micro-expression | APA – no entry |  |
|  | .. a facial expression that only lasts for a short moment. It is the innate result of a voluntary and an involuntary emotional response occurring simultaneously and conflicting with one another and occurs when the amygdala responds appropriately to the stimuli that the individual experiences and the individual wishes to conceal this specific emotion. This results in the individual very briefly displaying their true emotions followed by a false emotional reaction | Svetieva and Frank (2016) |
| Adverse experience | APA – no entry |  |

# Supplemental Material B - Figure

Dysregulation of Hypothalamus, Pituitary and Adrenal (HPA)- axis


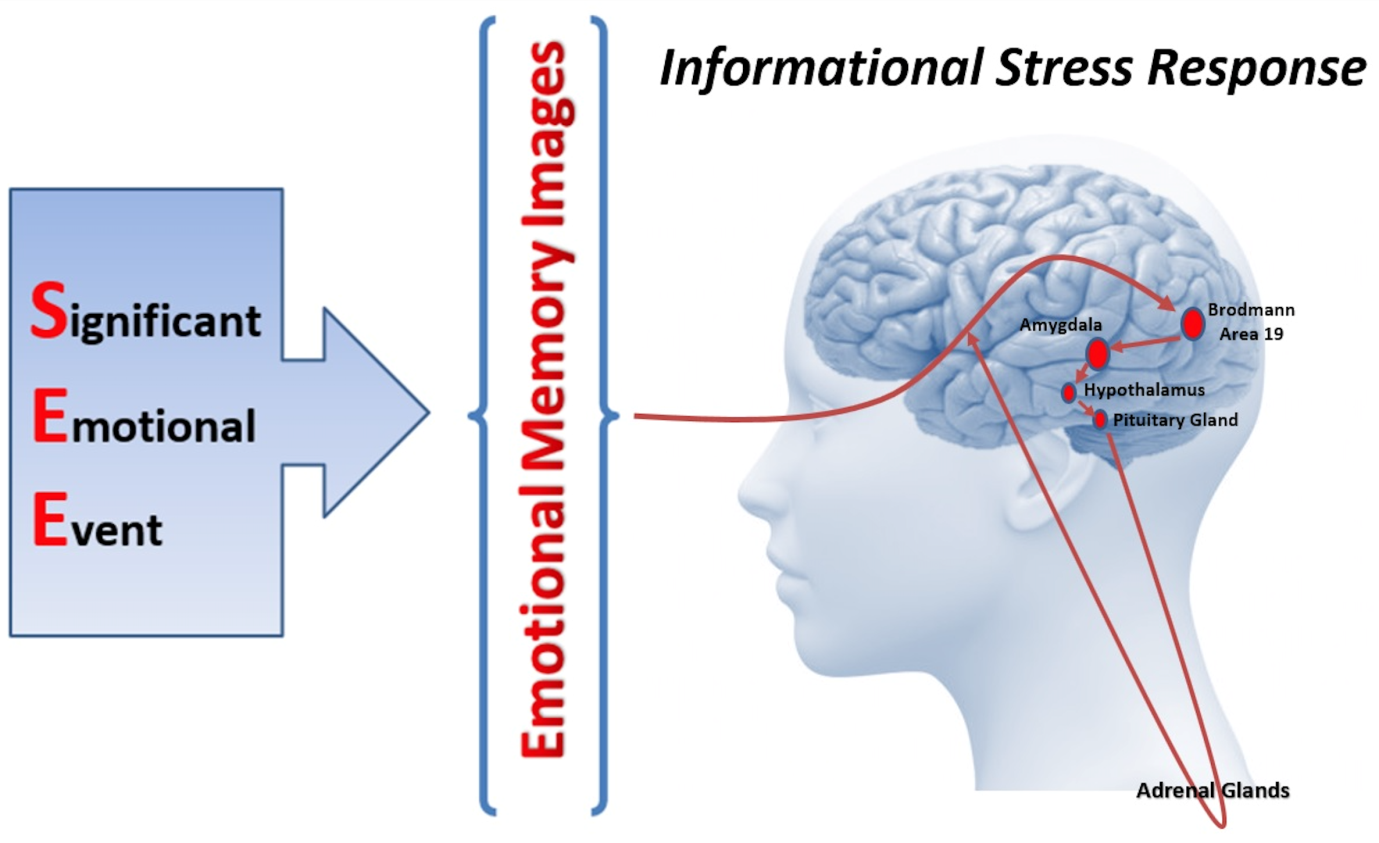

Supplement: Supplementary file 1 [file Table_1.DOCX]
